# The use of conspicuity aids by cyclists and risk of crashes involving other road users: a protocol for a population based case-control study

**DOI:** 10.1186/1471-2458-10-39

**Published:** 2010-01-27

**Authors:** Philip D Miller, Denise Kendrick, Carol Coupland, Frank Coffey

**Affiliations:** 1 Division of Primary Care, School of Community Health Sciences, University of Nottingham, The Tower, University Park, Nottingham, NG7 2RD, UK; 2 Emergency Department, Nottingham University Hospitals NHS Trust, Derby Road, Nottingham, NG7 2UH, UK

## Abstract

**Background:**

Regular cycling has been shown to improve health and has a role in tackling the threats posed by obesity and inactivity. Cycle collisions, particularly those involving motorised vehicles, can lead to significant mortality and morbidity and are currently a barrier to wider uptake of cycling. There is evidence that the conspicuity of cyclists is a factor in many injury collisions. Low-cost, easy to use retro-reflective and fluorescent clothing and accessories ('conspicuity aids') are available. Their effectiveness in reducing cycling collisions is unknown. The study is designed to investigate the relationship between the use of conspicuity aids and risk of collision or evasion crashes for utility and commuter cyclists in the UK.

**Methods/Design:**

A matched case-control study is proposed. Cases are adult commuter and utility cyclists involved in a crash resulting from a collision or attempted evasion of a collision with another road user recruited at a UK emergency department. Controls are commuter and utility cyclists matched by journey purpose, time and day of travel and geographical area recruited at public and private cycle parking sites. Data on the use of conspicuity aids, crash circumstances, demographics, cycling experience, safety equipment use, journey characteristics and route will be collected using self-completed questionnaires and maps. Conditional logistic regression will be used to calculate adjusted odds ratios and 95% confidence intervals of the risk of a crash when using any item of fluorescent or reflective clothing or equipment.

**Discussion:**

This study will provide information on the effectiveness of conspicuity aids in reducing the risk of injury to cyclists resulting from crashes involving other road users.

## Background

Regular cycling has the potential to help deliver important public health policy objectives such as reducing obesity and physical inactivity [1-5]. Modal shift from private car to bicycle could also help alleviate the problems of urban congestion and degradation of the environment [6,7]. There is considerable scope for expansion of cycling in many parts of the UK [8].

The risk of injury from bicycle crashes acts as a barrier against modal shift to cycling [9,10]. Targets to reduce injury rates among road users have been set by the UK government [11] and the safety of 'vulnerable road users' such as cyclists is of increasing concern internationally [12,13]. However recent data suggests that progress on reducing cycling crashes may have slowed [14].

Both epidemiological evidence and forensic engineering analyses suggest that low conspicuity is a factor in many cycling crashes. Late detection of other road users has been called "the fundamental driver error" leading to collisions [15]. An in-depth study of bicycle-car collisions found only 51% of car drivers had noticed the cyclist prior to their collision [16]. Drivers have difficulty detecting 'small' objects such as cyclists and pedestrians, especially at lower light levels or in darkness [17-19]. Richter et al [20] found 17.5% of cycling crashes occurred during dawn, dusk or at night. Despite the relatively small proportion of cycling crashes occurring at times of low visibility and bicycle use being lower at these times, a higher proportion of these incidents result in severe or fatal injury [21,22]. A study of cyclist fatalities in London (UK) found 41% occurred in darkness [23]. Recognition of cyclists seems particularly poor when motor vehicles are pulling alongside or approaching from behind [18]. A study in Victoria (Australia) found 90% of cyclist fatalities involved a rear impact suggesting that many drivers are unaware of the presence of a cyclist prior to a collision [24]. Adverse weather conditions appear to have only a minimal role in most bicycle crashes [25] but this finding may be explained by lower rates of cycle use in bad weather. If cycling were more widely adopted, including in adverse weather conditions, the importance of this factor in cycling crashes may increase.

The effect of conspicuity aids on cyclist crash risk is unknown [26]. Early experimental work demonstrated that simple conspicuity enhancing devices increase the distance over which drivers can detect and recognise cyclists and pedestrians [27-29]. Minimum performance requirements for such devices are important as drivers have a limited time to react to the presence of a cyclist particularly at higher speeds, in poor weather or under reduced ambient light [30].

Low-cost conspicuity aids suitable for the needs of non-competitive cyclists are readily available. Reflective or fluorescent materials can be worn as garments, on a helmet or attached to the bicycle, rider or luggage. Combinations of fluorescent and reflective materials also enhance conspicuity regardless of changes in visibility conditions occurring during a journey e.g. during dawn or dusk or on longer journeys where weather conditions may change. The use of 'biomotion' aids to emphasise the distinctive motion of a cyclist (e.g. pedal reflectors, spoke reflectors and fluorescent or reflective cycle clips or 'slap wraps') can also increase recognition distances [26]. Pedal reflectors have been mandatory on all bicycles sold in the UK since 1985. The UK Highway Code http://www.direct.gov.uk recommends the use of "high-visibility" clothing in addition to front and rear lights and rear reflectors which are mandatory under the Road Vehicles Lighting Regulations 1989. Many other countries have similar recommendations and legislation. Despite this, little is known about the current levels and patterns of use of conspicuity aids in many jurisdictions. An observational study of conspicuity aid use by cyclists and pedestrians in Edmonton (Alberta, Canada) found fewer than one third wearing outer clothing rated as 'medium' or 'high' visibility [31] and similarly low rates of conspicuity aid and light use were found in fatal cycling collisions in Australia [24]. An observational study in Oxford (UK) found 10% of cyclists used high-visibility clothing and less than half used lights at dusk and after dark [32]. A study of cyclists conducted in Paris and Boston (US) found great variation in the use of safety equipment such as lights between the two countries but in neither setting was the observed prevalence greater than 50% [33].

Cyclists' attitudes towards the use of conspicuity aids, in particular their beliefs as to what 'works' and under what conditions, could be a significant factor in understanding low adoption rates. Recent research by Wood and Lacherez [34] suggests that some cyclists overestimate the distance at which they would be noticed by drivers at night whilst underestimating the conspicuity enhancement offered by fluorescent or retro-reflective vests in darkness or daylight. These findings suggest that evidence of the effectiveness of conspicuity aids in reducing collision risk may help encourage greater use of conspicuity aids. There is no published research on the association between conspicuity aid use and risk of injury crash in cyclists. This protocol describes a case-control study to examine the relationship between conspicuity aid use and risk of cycling injuries.

## Methods/Design and Discussion

The study was designed to investigate the relationship between the use of conspicuity aids and the risk of an injury resulting from a collision with another road user or as a result of evasion to avoid such a collision ('collision or evasion crash', CEC) for commuter and utility cyclists.

The study was designed to achieve the following secondary objectives:

(a) to assess the effect of use of specific fluorescent and reflective items, bicycle lights, reflectors and clothing and helmet colour on risk of CEC

(b) to compare the effect of use of conspicuity aids on risk of CEC by light levels, weather conditions, gender, age and cycling experience.

A matched case-control design will be used. Data on demographic details, journey characteristics, exposures and confounding factors will be ascertained by self-completed questionnaires. Self-reported conspicuity aid and cycle helmet use will be validated by independent observation and measurement of inter-observer agreement.

Cases will be recruited from the Emergency Department (ED) or hospital wards of the Nottingham University Hospitals NHS Trust. Cases will be defined as all cyclists aged 16 years or over, commuting or undertaking a utility trip who are involved in a CEC within the study catchment area (the catchment area of Nottingham University Hospitals NHS Trust ED) and who attend the study site ED for assessment and/or treatment.

Commuter cyclists are defined as those making a journey to or from their place of work or study or on a work or study related trip or cycling a stage in any such journey e.g. to or from a railway station as part of a longer journey. Utility cyclists are defined as cyclists travelling for a purpose such as shopping who park or dismount in a public place at some point during their journey.

The study excludes cyclists travelling for the purposes of leisure, training or competition, those aged under 16 years, who lack the capacity to consent as a result of the crash (as assessed by either clinical staff or later by the researcher), who have been fatally injured, who are unwilling to give informed consent, whose crash occurs between 23:00 and 05:00 or whose crash occurs outside the study catchment area.

Controls will be recruited from cycle parking facilities at a sample of workplaces, colleges, universities, public cycle parking and transport facilities such as train stations and park and ride facilities, within the catchment area of Nottingham University Hospitals NHS Trust ED ensuring that case crash journeys and control journeys are undertaken within the same geographical area. Suitable workplaces will be identified using the Financial Analysis Made Easy (FAME) database of UK companies http://fame.bvdep.com. Written permission to recruit at their site will be gained from all organisations before recruitment activity is undertaken. Public cycle parking and cycle parking at transport facilities such as train stations and park and ride facilities will be identified using local government information services, maps and publications. Local Authority approval will be sought to allow permission to recruit at such sites. Further potential sites will be identified prospectively from the origin and destination data of cases recruited to the study.

Controls will be defined as cyclists aged 16 years or above commuting or undertaking a utility trip and will be approached once they have dismounted at the beginning or end of their journey, at a sample of cycle parking facilities at workplaces, colleges, universities, public cycle parking and cycle parking at transport facilities.

The study excludes cyclist controls if they are travelling for the purposes of leisure, training or competition, aged under 16 years, who are unwilling to give informed consent, who are travelling after 22:00 and before 06:00 and where most or all of their journey is undertaken outside the study catchment area.

An average of 4 controls per case will be recruited with the ratio of cases to controls allowed to vary from 1:1 to 1:6 to reflect the potential difficulty of recruiting controls at some sites or times. Controls will be individually matched to each case as follows:

• By day of week and season (control journey on the same weekday up to six weeks after the case's crash)

• By time of crash (any part of control journey within +/- one hour of the case's crash)

• By purpose of journey

Pilot data were collected (PM and DK) to establish the exposure levels for conspicuity aid use at five bicycle crash sites in the proposed study catchment area. Anonymised details of the crash site (grid reference and description), day and time of the crashes were supplied by the Vehicle Safety Research Centre at Loughborough University from their existing road crash investigation database ('On The Spot' Crash Investigation http://www.ukots.org/index.html). The number and conspicuity aid use of all cyclists passing each site for one hour including the time of day of the crash was recorded including the numbers using reflective garments, fluorescent garments, lights, reflectors and reflective or fluorescent equipment (such as pannier bags). The proportion of cyclists using any fluorescent or reflective clothing or item (excluding reflectors mandated by law) during peak times (07:30 to 09:30) was then calculated to be 44% (28 of 64).

To detect an odds ratio of 0.63 for CEC (derived from [35]) based on a prevalence of wearing or using any reflective or fluorescent clothing or items (excluding reflectors mandated by law) of 44%, a case-control correlation of 0.2 and a ratio of 1:4 cases to controls, 218 cases are required to give the study 80% power (2-sided α = 0.05).

Exposures will be recorded by participants using a self-completion questionnaire (Additional File 1). Cases will be asked to record their crash journey and controls the matched journey on the day they are given the study pack on simplified maps of the road network within the study catchment area.

Conspicuity aid details to be recorded will include;

• Any fluorescent material as part of upper body clothing, lower body clothing or cycle helmet

• Any retro-reflective material as part of upper body clothing, lower body clothing or cycle helmet

• Any light-coloured material as part of upper body clothing, lower body clothing or cycle helmet

• Reflectors mounted on the bike, pedals or spokes

• Fluorescent or reflective ankle bands or cycle clips

• Use of front and/or rear lights and whether lit, flashing or not lit.

• Any other safety equipment or accessories (e.g. fluorescent or reflective areas on back-packs or panniers)

The researcher (PM) will independently collect data on upper and lower body clothing and helmets ('fluorescent' and/or 'reflective' and/or 'mainly light-coloured') as a 'gold standard' to validate self-reported data. Assuming a 44% prevalence of conspicuity aid use (from the observations discussed above), a sub-sample of 140 participants (approximately 70 cases and 70 controls) will give an estimate of sensitivity of 80% with a 95% confidence interval width of +/- 10%.

Sunset, sunrise and twilight times will be obtained for Nottingham for each participant's journey date from http://www.timeanddate.com. For the purposes of classifying journey times for this analysis 'darkness' is defined as between the end of twilight in the evening of a given day and the beginning of twilight the following morning. 'Dawn' is defined as occurring from twilight in the morning until one hour after sunrise and 'dusk' is defined as one hour preceding sunset until twilight time in the evening. Extending the twilight category by one hour into daylight reflects the deterioration in visibility conditions as a result of low-angled sun and significant shading during these periods. 'Daylight' is defined as between one hour after sunrise to one hour prior to sunset.

Case journeys will be classified by the crash time falling within the periods defined above. Weather conditions for case and control journeys will be categorised using questionnaire responses (given in brackets) as 'Good' ('Good'), 'Moderate' ('Light Rain') or 'Poor' ('Heavy Rain', 'Fog/Mist' 'Snow/Hail') and self reported light levels will be categorised as reported ('Sunny', 'Overcast', 'Dawn/Dusk', 'Dark (street lights)' and 'Dark (no street lights)'.

Age and gender will be included in multivariate models as *a priori *confounders of the association of interest. Further potential confounding variables have been identified from the road crash research literature. Deprivation is a potential confounding variables as a study of fatal passenger vehicle crashes and deprivation found that high risk factors (e.g. excessive speed) were associated with increasing deprivation [36]. Noland and Quddus found a significant association between deprivation and injury severity for pedestrians although they lacked sufficient power to detect such a relationship for cycling injuries [37]. Deprivation scores (Index of Multiple Deprivation 2007; Social Disadvantage Research Centre, University of Oxford) for participants will be obtained using the post code of the home address matched to Lower Super Output Area data using GeoConvert software http://geoconvert.mimas.ac.uk/.

Risky road behavior may confound the effect of the study exposures. Risk taking cannot be measured directly and therefore proxy psychometric scales have been selected from the literature. 'Sensation Seeking' will be measured using the four item scale developed and validated by Stephenson and Hoyle for use in long surveys [38]. This trait has been shown to correlate with risky health behaviours (op. cit.) and to be associated with crash involvement in young drivers [39]. A four item scale developed by Kohn and Schooler [[40] in [41]] will be used to measure 'Normlessness'. This trait has been defined as a "belief that socially unapproved behaviours are required to achieve certain goals" and has been found to be associated with self reported risky on-road behavior in young drivers [41]. Cronbach's alpha will be used to measure the internal consistency of the data for both scales. The overall mean value of each four item scale will be calculated for each respondent. These scores will be included in the analysis as continuous covariates or dichotomised at the median if there is evidence of non-linearity.

Data will be collected for each participant's route. The number of injury pedal cycle crashes occurring in the previous three years (Department for Transport data), the numbers of cyclists passing randomly selected crash sites (from observations) and route length will be calculated. These variables will be used to control for potential confounding from both objective and perceived route risk. Increasing numbers of cyclists are known to reduce the rate of cycling crashes [42]. Features of the road environment are known to increase cyclists' perceptions of risk [43,44] which may affect both their road behavior and their use of conspicuity aids.

The use of reflectors and lights may confound the association between use of conspicuity aids and crash risk. The use of reflectors will be categorised as 'use of fixed reflectors' (front and/or rear reflector mounted on the bicycle), 'use of moving reflectors' (pedal reflectors and/or spoke reflectors and/or reflective ankle bands or clips) vs. 'none'. The use of lights (whether flashing or constant) will be categorised as 'use of front and rear mounted lights', 'use of front mounted light', 'use of rear mounted light' vs. 'none'.

Weather and light conditions are potential confounders as they may independently alter the risk of bicycle crashes. The amount of cycling experience of each participant may be a potential confounder for crash risk and will be recorded as 'less than one year', 'one to three years', 'four to ten years' and 'more than ten years' of regular cycling (one or more journeys per week) as an adult. The use of bicycle helmets has been shown to be associated with reduced injury severity of non-head-injured cyclists [45] and may therefore act as a further confounder for crash risk.

Response bias will be assessed for cases and controls. Responder and non-responder cases will be compared by age, gender and deprivation score (Index of Multiple Deprivation 2007) using a chi-squared test for gender and t-tests for differences in mean age and deprivation scores if these variables are normally distributed or the Mann-Whitney U test if not. Responder and non-responder controls will be compared by gender and independently recorded use of conspicuity aids and helmets using the chi-squared test.

Continuous data will be described separately for cases and controls using means and standard deviations, or medians and inter-quartile ranges, dependant on the normality of the distributions. Categorical data will be described in cases and controls using frequencies and percentages.

Odds ratios and 95% Confidence Intervals will be estimated using conditional logistic regression modeling. The primary measure of interest will be the odds ratio for being involved in a CEC whilst using any item of fluorescent or reflective clothing or equipment (in addition to reflectors mandated by law) compared with none. The analysis will adjust for the *a priori *and potential confounders described above. Potential confounders will be included in multivariate models if they alter the estimate of the odds ratio for the main exposure variable by more than 10% [46]. Significance testing will be based on likelihood ratio tests with a p value of < 0.05 taken as statistically significant.

Differences in the effectiveness of conspicuity aids by injury severity will be assessed by estimating odds ratios separately for cases with different levels of injury severity and their matched controls. Differences in the effectiveness of conspicuity aids by light levels, weather conditions, route risk, gender, age and cycling experience will be explored by adding interaction terms to the multivariable regression models. Where significant interactions (p < 0.05) are found odds ratios for sub-groups will be reported separately.

Validation data will be compared by calculating kappa coefficients with 95% confidence intervals for sets of paired independent observations.

All analyses will be conducted using STATA version 10 (StataCorp LP, Texas, US).

The study has been reviewed and approved by the North Nottinghamshire NHS Research Ethics Committee (Ref: 07/H0407/81) the Nottingham University Hospitals NHS Trust Research and Development department (Ref: 07AE003) and the research governance system of the study sponsor the University of Nottingham.

All cases will provide written consent for the use of their data and for the researcher to access collect data from the clinical records of their injuries. A consent form (included in the study questionnaire) will be signed and dated by each participant before they are entered into the study. Return of a completed questionnaire by controls will be presumed to constitute consent to participate in the study.

Cases who return a completed questionnaire (whether eligible for inclusion in the primary analysis or not) will be sent a £5 shopping voucher to compensate them for their time spent completing and returning the study instrument.

## Abbreviations

CEC: Collision or Evasion Crash; ED: Emergency Department.

## Competing interests

The authors declare that they have no competing interests.

## Authors' contributions

PM developed the detailed protocol and study instruments, is responsible for recruitment and analysis and has written the manuscript. DK and CC proposed the original study concept, have co-authored the protocol, are supervising the conduct and analysis of the study and the academic training of PM and have edited the manuscript. FC is the local collaborator for the study and gave advice on the conduct of the study on NHS premises, commented on the protocol, reviewed the research ethics application and study documentation, is providing clinical supervision of the conduct of the study and has commented on the manuscript. All authors read and approved the final manuscript.

## Pre-publication history

The pre-publication history for this paper can be accessed here:

http://www.biomedcentral.com/1471-2458/10/39/prepub

## Supplementary Material

Additional file 1 **Case Self-Completion Questionnaire**. Case version of the RES-approved questionnaire for distribution to potential participants after they have attended the study site emergency department. Click here for file
