# Supplementary material for: The use of conspicuity aids by cyclists and risk of crashes involving other road users: a protocol for a population based case-control study
Source: BMC Public Health. 2010 Jan 27;10:39. doi: 10.1186/1471-2458-10-39 (PMC2835683; doi:10.1186/1471-2458-10-39)
Supplement: Additional file 1 — Case Self-Completion Questionnaire. Case version of the RES-approved questionnaire for distribution to potential participants after they have attended the study site emergency department. [file 1471-2458-10-39-S1.DOC]

| 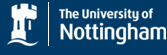 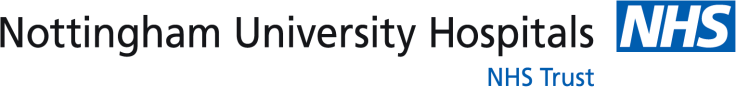 |
| --- |

| Nottingham Bicycle Accident Study  Thank you for taking part in this research project. As you will have seen from the information provided in this pack the study is an investigation of factors which may affect the risk of having a bicycle accident.  We would like you to complete the questionnaire and journey map and return them to us in the stamped, addressed envelope provided. It is important that you fill in the questionnaire as soon as you feel able to so that you can accurately recall things as they were on the day you had your accident. The questionnaire should take no longer than 10 minutes to complete.  Please remember that this is a research project and not connected to your hospital treatment. Because of this we would like you to sign the consent form to say that we can use your information. This can be found on the final page of this booklet.  Completing this questionnaire and returning it in the stamped address envelope is all that you will be asked to do to support this project. You will not be contacted again unless you require different maps to draw your journey or request a summary of the results.  If you would like to receive a summary of the results of this research please tick the box on the consent form at the end of the questionnaire.  If you require any further information, please contact the study organiser;  Phil Miller  PhD student researcher  School of Community Health Sciences  Room 1401, The Tower  University of Nottingham  Nottingham  NG7 2RD  0115 8230576  [mcxpdm@nottingham.ac.uk](mailto:mcxpdm@nottingham.ac.uk)  Thank you | |
| --- | --- |
| Study ID (Office Use Only) |  |
| Name: ­­­­­­­­­­­­­­­­­­­­­­­­­­­­­­_________________________________________  Home Address: __________________________________    _______________________________________________  _______________________________________________  Postcode: _______________________________________  Contact telephone: ­­­­­­­­­­­­­­­­­­­­­­­­­­­________________________________ | |

| QUESTIONS ABOUT YOUR ACCIDENT JOURNEY |
| --- |

| Please answer the following questions about your accident journey  What time did your accident happen? ___ : ___ (24 hour clock)  What was the date of your accident? ___ / ___ / ______ (DD/MM/YYYY) | | | | | | | | | | | | | | | | | |
| --- | --- | --- | --- | --- | --- | --- | --- | --- | --- | --- | --- | --- | --- | --- | --- | --- | --- |
| Where did you start your accident journey (full address and postcode if known)? If this is a workplace please include the company or organisation name. | | | | | | | | | | | | | | | | | |
| Please tick ONE only | | | | | | | | | | | | | | | | | |
| Is this  (a) a public place (e.g. shops, libraries, a cycle trail etc)?  | | | | | | | | | | | | | | | | | |
| (b) a workplace, college or university?  | | | | | | | | | | | | | | | | | |
| (c) a private address (e.g. your home or a friend’s house etc)?  | | | | | | | | | | | | | | | | | |
| (d) cycle parking at transport facilities (e.g. train station, park and  ride etc) ?   (e) other place (please describe)?   Where were you intending to finish your journey (full address and postcode if known)? If this is a workplace please include the company or organisation name. | | | | | | | | | | | | | | | | | |
| Please tick ONE only | | | | | | | | | | | | | | | | | |
| Is this (a) a public place (e.g. shops, libraries, a cycle trail, etc)?  | | | | | | | | | | | | | | | | | |
| (b) a workplace, college or university?  | | | | | | | | | | | | | | | | | |
| (c) a private address (e.g. your home or a friend’s house etc)?  | | | | | | | | | | | | | | | | | |
| d) cycle parking at transport facilities (e.g. train station, park and  ride etc) ?   (e) other place (please describe) ?   What was the purpose of the journey during which you had your accident?  (for example travelling to work or college, shopping, visiting a friend, leisure ride etc) | | | | | | | | | | | | | | | | | |
| Please draw on the map provided the complete journey you would have made had you not had your accident. Please put a cross at the spot where your accident happened. | | | | | | | | | | | | | | | | | |
| If you need different maps I will send you ones including your start and finish points. Even if you need different maps, please fill in the rest of this questionnaire.  Please send me maps including these start and finish points  | | | | | | | | | | | | | | | | | |
| Please describe the location of the accident as clearly as possible - include any details you are aware of like nearby shops or landmarks (e.g. “I was cycling along the road through the pelican crossing on Huntingdon Street near the Victoria Centre car park exit”) | | | | | | | | | | | | | | | | | |
| If you started or intended to finish your journey at a workplace, college, university or transport facility | | | | | | | | | | | | | | | | | |
| Are cycle changing facilities  and lockers provided at this location? | | | | | Yes | |  | No | |  | Don’t know | |  | | Not Applicable | |  |
| Are cycle parking facilities e.g. Sheffield hoops provided at this location? | | | | | Yes | |  | No | |  | Don’t know | |  | | Not Applicable | |  |
| Do you feel that this organisation actively encourages you to cycle? | | | | | Yes | |  | No | |  | Don’t know | |  | | Not Applicable | |  |
| If you journey was to or from a workplace  How many employees does your company have? | | | | | | | | | | | | | | | | | |
| Less than  50 |  | 50 to  250 |  | More than  250 | |  | | | Don’t Know | | |  | | N/A | |  | |

| QUESTIONS ABOUT YOUR ACCIDENT |
| --- |

| Did your accident involve a collision with another road user (e.g. car, motorcycle, cyclist, bus, heavy goods vehicle, tram or pedestrian)? | | | | | | | | | | | | | | | | | | | | | | | | | | | | Yes | | | | |  | | | | | | | No | | | |  | | | | |
| --- | --- | --- | --- | --- | --- | --- | --- | --- | --- | --- | --- | --- | --- | --- | --- | --- | --- | --- | --- | --- | --- | --- | --- | --- | --- | --- | --- | --- | --- | --- | --- | --- | --- | --- | --- | --- | --- | --- | --- | --- | --- | --- | --- | --- | --- | --- | --- | --- |
| Was your accident caused when trying to avoid a collision with another road user (e.g. car, motorcycle, cyclist, bus, heavy goods vehicle, tram or pedestrian)? | | | | | | | | | | | | | | | | | | | | | | | | | | | | Yes | | | | |  | | | | | | | No | | | |  | | | | |
| Where were you when the accident happened e.g. on the pavement or cycle path or on the road?  Please tick ONE only | | | | | | | | | | | | | | | | | | | | | | | | | | | | | | | | | | | | | | | | | | | | | | | | |
| Foot path / pavement | | | | | | | | | | | | | | | | | | | | | | | | | | | | | | | | |  | | | | | | | | | | | | | | | |
| Cycle path (separate from the road but including paths shared with pedestrians) | | | | | | | | | | | | | | | | | | | | | | | | | | | | | | | | |  | | | | | | | | | | | | | | | |
| Side road / residential street (including cycle lanes on the road) | | | | | | | | | | | | | | | | | | | | | | | | | | | | | | | | |  | | | | | | | | | | | | | | | |
| Main road / through road (including cycle lanes on the road) | | | | | | | | | | | | | | | | | | | | | | | | | | | | | | | | |  | | | | | | | | | | | | | | | |
| Other (please describe)  | | | | | | | | | | | | | | | | | | | | | | | | | | | | | | | | | | | | | | | | | | | | | | | | |
| Were you at or within 20 metres (60 feet) of a junction e.g. cycle path joining a road or a roundabout or a T-junction? | | | | | | | | | | | | | | | | | | | | | | | | | | | | Yes | | | | |  | | | | | | | No | | | |  | | | | |
| What was the speed limit of the road where your accident occurred (mph)?  Please tick ONE box | | | | | | | | | | | | | | | | | | | | | | | | | | | | | | | | | | | | | | | | | | | | | | | | |
| 20 |  | | 30 | | | |  | | | 40 | | |  | | | 50 | |  | | 60 | | | |  | | | 70 | |  | | | | | Not on a road | | | | | | |  | | Don’t know | | | | |  |
| How often have you cycled on this route in the past 6 months?  Please tick ONE box | | | | | | | | | | | | | | | | | | | | | | | | | | | | | | | | | | | | | | | | | | | | | | | | |
| More than 2 times a week? | | | | | |  | | | Between two and eight times a month? | | | | | | | | | |  | | Less than once a month? | | | | | | | | | |  | | | | | Never before? | | | | | | | | | |  | | |
|  | | | | | | | | | | | | | | | | | | | | | | | | | | | | | | | | | | | | | | | | | | | | | | | | |
| Had you drunk any alcohol in the 8 hours prior to this journey? | | | | | | | | | | | | | | | | | | | | | | | | | | | | | | | | Yes | | | | | |  | | | | No | | |  | | | |
|  | | | | | | | | | | | | | | | | | | | | | | | | | | | | | | | | | | | | | | | | | | | | | | | | |
| How would you describe the weather conditions at the time of your accident?  Please tick ONE box | | | | | | | | | | | | | | | | | | | | | | | | | | | | | | | | | | | | | | | | | | | | | | | | |
| Good weather | | | |  | | | | Light rain | | | | | | |  | | Heavy rain | | | | | |  | | | Fog/mist | | | | | | | | |  | | | Snow/hail | | | | | | | | |  | |
| How would you describe the light levels at the time of your accident?  Please tick ONE box | | | | | | | | | | | | | | | | | | | | | | | | | | | | | | | | | | | | | | | | | | | | | | | | |
| Sunny | |  | | | Overcast | | | | | | |  | | Dawn/  dusk | | | | |  | | | Dark (no street lights) | | | | | | | |  | | | | | Dark (street lights) | | | | | | | | | | | |  | |
|  | | | | | | | | | | | | | | | | | | | | | | | | | | | | | | | | | | | | | | | | | | | | | | | | |
| What type of bicycle were you riding when you had your accident?  Please tick ONE box | | | | | | | | | | | | | | | | | | | | | | | | | | | | | | | | | | | | | | | | | | | | | | | | |
| Commuter or ‘hybrid’ or city bike | | | | | | | | | | |  | | | | Mountain or ‘off road’ bike | | | | | | |  | | | Road or racing bike | | | | | | | | | | | |  | | Folding bike | | | | | | | |  | |
| If you used another type of bike please describe it in the space below | | | | | | | | | | | | | | | | | | | | | | | | | | | | | | | | | | | | | | | | | | | | | | | | |

| QUESTIONS ABOUT YOUR CYLING EQUIPMENT AND CLOTHING |
| --- |

| The following questions are about what equipment you were using, what you were carrying and what you were wearing when you had your accident | | | | | | | | | | | | |
| --- | --- | --- | --- | --- | --- | --- | --- | --- | --- | --- | --- | --- |
| Were you wearing a CYCLE HELMET? | | | | | | Yes |  | | No | |  | |
| If yes, was most of the helmet made from FLUORESCENT materials?  (usually bright orange, yellow or lime green) | | | | | | Yes |  | | No | |  | |
| Is your helmet a LIGHT COLOUR (e.g. white or yellow)? | | | | | | Yes |  | | No | |  | |
| Does your helmet have any REFLECTIVE areas e.g. panels or edging?  (usually white or silver) | | | | | | Yes |  | | No | |  | |
| Please describe the OUTER clothing you were wearing ABOVE THE WAIST  in the space below (e.g. tabard or jacket) | | | | | | | | | | | | |
| Was most or all of this garment LIGHT COLOURED? | | | | | | Yes |  | | No | |  | |
| Was most or all of this garment made from FLUORESCENT materials?  (usually bright orange, yellow or lime green) | | | | | | Yes |  | | No | |  | |
| Did this garment have any REFLECTIVE areas e.g. panels or edging?  (usually white or silver) | | | | | | Yes |  | | No | |  | |
| Please describe the OUTER clothing you were wearing BELOW THE WAIST  in the space below (e.g. trousers or shorts) | | | | | | | | | | | | |
| Was most or all of this garment LIGHT COLOURED? | | | | | | Yes |  | | No | |  | |
| Was most of this garment made from FLUORESCENT materials?  (usually bright orange, yellow or lime green) | | | | | | Yes |  | | No | |  | |
| Did this garment have any REFLECTIVE areas e.g. panels or edging?  (usually white or silver) | | | | | | Yes |  | | No | |  | |
| Did you have PEDAL REFLECTORS? | | | | | | Yes |  | | No | |  | |
| Did you have FLUORESCENT ankle bands or bicycle clips? | | | | | | Yes |  | | No | |  | |
| Did you have REFLECTIVE ankle bands or bicycle clips? | | | | | | Yes |  | | No | |  | |
| Did you have a FRONT FACING REFLECTOR(s)? | | | | | | Yes |  | | No | |  | |
| Did you have a REAR FACING REFLECTOR(s)?  (including on panniers or saddle bag) | | | | | | Yes |  | | No | |  | |
| Did you have SPOKE OR WHEEL REFLECTORS? | | | | | | Yes |  | | No | |  | |
| Did you have a FRONT FACING LIGHT(s)? | Yes, it was lit |  | Yes, it was flashing |  | Yes, but it  was not lit | | |  | | No | |  |
| Did you have a REAR FACING LIGHT(s)? | Yes, it was lit |  | Yes, it was flashing |  | Yes, but it was not lit | | |  | | No | |  |
| Please describe any other safety equipment you were using on the day of your accident that has not been mentioned, in the space below | | | | | | | | | | | | |

| QUESTIONS ABOUT YOU |
| --- |

| The following questions are about you | | | | |
| --- | --- | --- | --- | --- |
| How old are you? | Years |  |  | |
| What gender are you? | Male |  | Female |  |
| Do you have a full driving license?  (including motorcycle, HGV etc) | Yes |  | No |  |

| What is your ethnic group? (choose ONE from section A to E then tick the appropriate box)  A White | | | | | | | | | | | | | | | | | | | |
| --- | --- | --- | --- | --- | --- | --- | --- | --- | --- | --- | --- | --- | --- | --- | --- | --- | --- | --- | --- |
| British | |  | | | Irish | |  | | | | Any other White background please  write | | | |  | | | | |
| B Black or Black British | | | | | | | | | | | | | | | | | | | |
| Caribbean | | |  | African | | | |  | | | | Any other Black Background  please  write | | |  | | | | |
| C Mixed | | | | | | | | | | | | | | | | | | | |
| White and Black Caribbean | | | | | |  | | | White and Black African | | | | |  | | White and Asian | | |  |
| Any other mixed background  please write | | | | | | | | | | | | | | |  | | | | |
| D Asian | | | | | | | | | | | | | | | | | | | |
| Indian |  | | | | | | | | | Pakistani | | |  | | | | Bangladeshi |  | |

| Any other Asian background please write | | |  |
| --- | --- | --- | --- |
| E Chinese or other ethnic group | | | |
| Chinese |  | Any other please write |  |

| QUESTIONS ABOUT YOUR BICYCLE USE |
| --- |

| How long have you been cycling regularly as an adult?  (one or more journeys per week on average) | | | | | | | | | | |
| --- | --- | --- | --- | --- | --- | --- | --- | --- | --- | --- |
| Less than one year? |  | One to three years? |  | Four to ten years? |  | | More than ten years? | | |  |
|  | | | | | | | | | | |
|  | | | | | | Yes | | No | Don’t  know | |
| Did you receive any formal cycle ‘proficiency’ training whilst at school? | | | | | |  | |  |  | |
| Have you had any formal cycle training after leaving school? | | | | | |  | |  |  | |
| Have you had a bicycle accident in which you were injured within the past 3 years involving a collision or ‘near miss’ with another road user? | | | | | |  | |  |  | |

| How many times have you ridden your bicycle in the 7 days prior to your accident? | Number of journeys | |  | |
| --- | --- | --- | --- | --- |
|  |  | |  | |
| How far have you travelled on your bicycle in the 7 days prior to your accident? |  | | Miles   Kilometres  | |
| Is this a typical amount of cycling for you? | Yes |  | No |  |

| QUESTIONS ABOUT YOUR ATTITUDES |
| --- |

| The following questions are about your attitudes in everyday life. These answers will help us to interpret the rest of the information you have given by filling in this survey.  Please try and answer them in a way which best reflects your own feelings and opinions – try not to think about them for too long – give the first answer that occurs to you. | | | | | |
| --- | --- | --- | --- | --- | --- |
|  | Strongly Agree | Agree | Neutral | Disagree | Strongly Disagree |
|  | Tick ONE box for each line | | | | |
| It is all right to do anything you want as long as you keep out of trouble |  |  |  |  |  |
| It is OK to get round  laws and rules as long as you don’t break them directly |  |  |  |  |  |
| If something works it is less important whether it is right or wrong |  |  |  |  |  |
| Some things can be wrong to do even though they are legal |  |  |  |  |  |
| I like to explore strange places |  |  |  |  |  |
| I like to do frightening things |  |  |  |  |  |
| I like new and exciting experiences, even if I have to break the rules |  |  |  |  |  |
| I prefer friends who are exciting and unpredictable. |  |  |  |  |  |

| QUESTIONS ABOUT YOUR EVERYDAY USE OF CLOTHING AND CYCLE EQUIPMENT |
| --- |

| The following questions are about your usual use of safety equipment and in what circumstances you normally use each item  Please tick all the times you use each type of equipment (e.g. if you use a front light after dark AND on long journeys tick BOTH of the boxes below these responses) | | | | | | | |
| --- | --- | --- | --- | --- | --- | --- | --- |
|  | Never | In bad weather | After dark | On long journeys | On fast roads | In heavy traffic | Always |
|  | Tick ALL that apply | | | | | | |
| When do you normally wear a CYCLE HELMET? |  |  |  |  |  |  |  |
| Do you wear any fluorescent or reflective clothing ABOVE THE WAIST  e.g. tabard or jacket |  |  |  |  |  |  |  |
| Do you wear any fluorescent or reflective clothing BELOW THE WAIST?  e.g. cycling shorts or leggings |  |  |  |  |  |  |  |
| Do you use fluorescent OR reflective ANKLE BANDS, WRAPS OR BICYCLE CLIPS |  |  |  |  |  |  |  |
| Do you use a FRONT FACING LIGHT? |  |  |  |  |  |  |  |
| Do you use a REAR FACING LIGHT? |  |  |  |  |  |  |  |
| Do you use any other cycling safety equipment we have not mentioned?  Please write the item in below and use the boxes to indicate when you use this item | | | | | | | |
|  | Never | In bad weather | After dark | On long journeys | On fast roads | In heavy traffic | Always |
|  |  |  |  |  |  |  |  |
|  |  |  |  |  |  |  |  |
|  |  |  |  |  |  |  |  |
| Thank you for completing this questionnaire. All information you provide will be treated confidentially and kept securely at the University of Nottingham. It will only be used for the purposes described in the information sheet.  Please complete the consent form on the next page as a record that you agree to take part. When this is done please return the complete form using the stamped addressed envelope provided for you.  Would you like to make any comments or give any further information? Please use the space below | | | | | | | |

| CONSENT  Study Number: 07/H0407/81  Name of Researcher: Phil Miller  Title of Project: Nottingham Bicycle Accident Study  Please initial  each box | | | | | | |
| --- | --- | --- | --- | --- | --- | --- |
| I confirm that I have read and understand the information sheet (case) dated 02/01/2008 Version 3.0 for this study. | | | |  | | |
| I understand that my participation is voluntary and that I am free to withdraw at any time, without giving any reason. | | | |  | | |
| I understand that parts of my emergency department clinical record will be accessed by members of staff from Nottingham University Hospitals and the University of Nottingham. | | | |  | | |
| I agree to take part in this study. | | | |  | | |
| _____________________ ________________ _____________________  Your Name Date Signature  _____________________ ________________ __­­___________________  Researcher Date Signature  Thank you for taking the time to complete this questionnaire – please return it in the stamped addressed envelope provided. A copy of this consent form will be sent to you and another placed in your medical records.  Phil Miller | | | | | | |
| Would you like to receive a summary of the results of this research? | | Yes |  | | No |  |
| Study ID |  | | | | | |
